# Optical Gas Imaging with Cooled and Uncooled Thermal Infrared Cameras †

**DOI:** 10.3390/s26103270

**Published:** 2026-05-21

**Authors:** Gabriel Jobert, Nicolas Vannier, Charlène Lefèvre, Eléa Bourliaud, Adrien Bertrand, Emmanuelle Chazelle, Eric Mallet

**Affiliations:** Lynred, 364 Route de Valence, 38113 Veurey-Voroize, France

**Keywords:** optical gas imaging, methane, infrared, thermal, cooled, uncooled, LDAR, visualization

## Abstract

In a context of greenhouse-gas-reduction for climate-change mitigation, Optical Gas Imaging (OGI) is cited by US and EU regulations as a key technology for detecting methane leaks in the oil and gas industry. The paper outlines the principles of OGI, covering specificity of both high-performance cooled cameras and cost-effective thermal infrared uncooled cameras. It explains camera design, the optical-radiometric theory of contrast and sensitivity, and provides a comprehensive description of the key performance indicators (KPIs) such as NETD, NECL, and MDLR; together with parameters that influence them. These theoretical concepts are supported by measurements taken under laboratory conditions and outdoors, with wind and complex scenes. Finally, video-processing methods for visualizing gas leaks are presented, showing how they increase visual sensitivity and reduce the user’s cognitive load.

## 1. Introduction

Methane (CH_4_), is a flammable gas widely used as a fuel for heating, power generation, and transport. Today we rely primarily on fossil methane, the main component of natural gas extracted from the earth. Bio-methane, obtained by harnessing the products from the decay of organic material such as agricultural waste, may offer a carbon-neutral alternative to fossil fuels [1].

All stages of methane handling within the oil and gas industry, from extraction, production to storage and distribution, generate fugitive emissions that represent a third of man-made methane emission [2]. This is especially critical knowing that CH_4_ is a potent greenhouse gas, having a GWP-20 (Global warming potential over 20 years) of 81.2 [3]. It means that one ton of atmospheric CH_4_ traps the same amount of heat as 81.2 tons of carbon dioxide (CO_2_) over 20 years. It is the second most influential greenhouse gas after CO_2_, accounting for 30% of global warming effects. Methane has an atmospheric lifetime of 8 years [4], and is often associated with short-lived climate forcers (SLCF). Tackling SLCF is a crucial part of climate change mitigation and can deliver significant near-term benefits [5].

In this context, regulatory bodies in both the United States (US) and the European Union (EU) have implemented new policies aimed at improving methane detection and mitigation. The US Environmental Protection Agency (US-EPA) has introduced regulations under the New Source Performance Standards (NSPS) Quad Os, which expand leak detection and repair (LDAR) requirements, explicitly citing Optical Gas Imaging (OGI) technology. The technical requirements for OGI cameras are detailed in Appendix K of the Final Rule (2022) [6]. It states notably the conditions in which a gas leak must be detected, citing: ‘*Must be capable of detecting (or producing a detectable image of) methane emissions of 19 g/h (...) at a viewing distance of 2.0 m and a ΔT of 5.0 °C in an environment of calm wind conditions around 1.0 m/s or less*’. The need to give a specific temperature differential and wind conditions highlights the influence of environmental factors on the detection capabilities of thermal imaging technologies, that we will discuss in this manuscript.

Concurrently, the European Union has enacted Regulation (EU) 2024/1787 [7], a legislative framework designed to enforce comprehensive LDAR programs, prohibit routine venting and flaring, and mandate greater transparency in methane emissions along fossil fuel supply chains. The EU regulation classifies LDAR requirements into two distinct categories. General-purpose leak detection: Type 1 LDAR requires a Minimal detectable leak rate (MDLR) of 17 g/h, which is similar to that of US-EPA regulation; Highly sensitive Type 2 LDAR requirements are 1 g/h MDLR, for high-risk infrastructure or critical emission sources. The EU regulation does not explicitly specify a gas-to-background temperature differential (ΔT) for methane detection.

Moreover, the Oil and Gas Methane Partnership 2.0 (OGMP 2.0) is a measurement-based reporting framework for methane emissions led by the United Nations Environment Programme [8]. OGMP 2.0 sets reporting levels from 1 to 5. Level 4 (L4) requires full inventory of potential sources, and estimated emissions through source level direct measurements. L5 expands on L4’s prior source inventory by estimating the site’s total emissions as the sum of every source-level quantitative measurement. This bottom-up approach is cross-checked against a site-level emission measurement, and the two results must be reconciled within their respective uncertainty. OGMP 2.0’s L4 and L5 requires the use of gas detection technologies, including OGI.

## 2. State-of-the-Art

### 2.1. IR Sensors for Optical Gas Imaging (OGI) Across Multiple Wavelengths

Optical Gas Imaging (OGI) is an industry standard, field-proven technology, widely used to detect, locate and quantify gas leaks. Among commercial OGI cameras from multiple vendors worldwide, we can cite Mileva 33 (cooled) and Caroline Y (uncooled) from SENSIA (Madrid, Spain); EyeCGas (cooled) from Opgal (Karmiel, Israel); GMP03 (cooled) from Konica-Minolta (Tokyo, Japan); GF77 (uncooled) and Gx620 (cooled) from FLIR Systems AB (Taby, Sweden); VENTUS OGI (cooled) and VIENTO LWIR OGI (uncooled) from Sierra-Olympia (Hood River, OR, USA) and several others. Lynred (Veurey-Voroize, France) is a historical IR sensor manufacturer and a major supplier for OGI camera manufacturers (part of those cited above).

**Figure 1:**
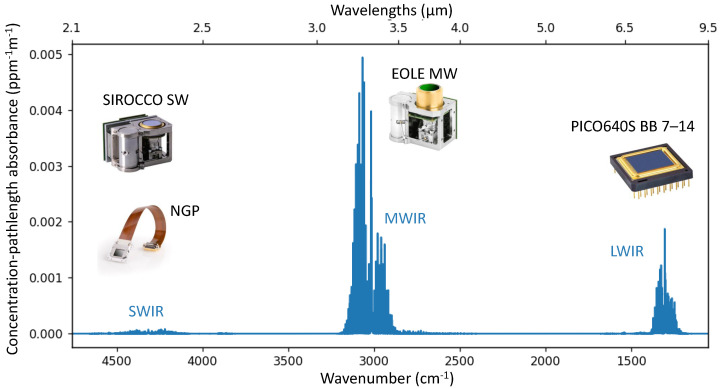
Concentration-pathlength absorbance spectrum of methane at 296 K air temperature and 1 atm air pressure, derived from HITRAN [9].

Such cameras rely on the absorption (and thermal emission) of gas molecules at a specific wavelength range in the IR. This allows to capture an image of the gas plume using special IR cameras, with a sensitivity that matches the spectral signatures of the targeted gas: CH_4_ in our case. In Figure 1, we plot its concentration-pathlength absorbance spectrum.

Methane has three main absorption bands: one in the Long-Wave IR (LWIR) range (7.2–8.3 μm), one in the Middle-Wave IR (MWIR) range (3.2–3.55 μm), and one in the Short-Wave IR (SWIR) range (2.31–2.39 μm).

**LWIR for specificity**: This absorption band, commonly known as ‘*fingerprint region*’, contains specific bending and wagging modes of the methane molecule. This allows for differentiation of methane from other hydrocarbons. To target this band, we consider the uncooled micro-bolometer array technology, and in this manuscript more specifically—Lynred’s PICO640S BB 7–14 that will be described further in Section 3. Micro-bolometer sensors are a cost-effective thermal imaging technology, fabricated using an above-CMOS (Complementary Metal Oxide Semiconductors) process similar to MEMS (Micro-Electro-Mechanical Systems) foundries. They benefit from decades of industrial development driven by high-volume markets, which have improved the performance-cost trade-off, with a sensitivity compatible with source-level inspections. Because it requires no active cooling, the sensor can operate reliably 24/7 for years, making it ideal for site monitoring in CEMS (Continuous Emission Monitoring Systems).

**MWIR for sensitivity**: This absorption band corresponds to the stretching modes of the carbon-hydrogen (C–H) molecular bond. This band has a high absorbance, is common for all hydrocarbons (HC), and is well fitted to detect faint HC gas leaks, such as CH_4_. Cooled IR image sensors have an integrated cold filter within the IDCA (Integrated Dewar Cooler Assembly) that is replaced by a narrow-band pass filter (3.2–3.55 μm band pass) that targets this HC band. In this study, we consider Lynred’s EOLE MW sensor, also described further in Section 3. Cooled MWIR sensors offer the most sensitive solution, well fitted for precise leak quantification at the source-level with a handheld camera, or carried on drones. Recent improvements in HOT (high operating temperature) sensor technologies and associated linear Stirling cryo-coolers enable reliable operating temperature up to 150 K with typical MTTF (mean-time-to-failure) specified at 27,000 h in a 24/7 operation life profile, which make it also quite fitting for CEMS.

**SWIR hyperspectral imaging for air and space**: This is the preferred band for airborne surveys [10] and satellite-based emission monitoring applications. Because of the lack of thermal radiation at this wavelength range, passive SWIR spectrometers enable spectral reflectance analysis from solar illumination. Those instruments usually rely on SWIR image sensors in a typical push-broom hyper-spectral imager (HSI) or FTIR (Fourier-Transform IR) setups. Those array sensors are usually made with HgCdTe photodiodes—Lynred’s legacy technology which includes spaceborne sensors such as NGP (Next-Generation Panchromatic) sensor: a 1 Mpix HgCdTe detector array covering the 0.8–2.5 μm range [11]; or IDCAs better suited for industrial applications such as SIROCCO SW: a VGA array which inherits from NGP’s photodiode technology [12].

Methane emission monitoring from space is performed by combining several satellites that carry instruments with very different characteristics. First, the ‘global flux mappers’ provide full earth coverage with a daily revisit rate. Examples include the European Space Agency’s (ESA) Copernicus S5P (Sentinel-5 Precursor) satellite that carries the TROPOMI instrument (TROPOspheric Monitoring Instrument) [13], or its successor Sentinel-5/UVNS (Ultraviolet-Visible-NIR-SWIR) [14] carried in ESA’s MetOp-SG satellite. Both intruments have a similar push-broom HSI that includes a SWIR band (2305–2385 nm) enabling measurement of methane concentrations in an atmospheric column with a ground sample distance (GSD) of 7 × 5.5 km, and a Limit of Detection (LoD) of roughly 200–250 kg/h of methane. This instrument is particularly effective at notifying stakeholders when an ultra-emitter emerges. Note that data from Copernicus satellites are openly available through the *Copernicus Atmosphere Monitoring Service* (CAMS) [15].

Second, ‘point-source imagers’ are instruments equipped with a high spatial resolution telescope. For example, the Italian Space Agency’s PRISMA (*PRecursore IperSpettrale della Missione Applicativa*) satellite provides a SWIR hyperspectral image (400–2500 nm) with a 30 m GSD, allowing a more precise targeting of an industrial site on demand.

Last, private new-space companies are developing constellations of micro-satellites dedicated to methane monitoring. For instance, the GHGSat [16] constellation (Montreal, QC, Canada) or the GESat [17] constellation from Absolut Sensing (Toulouse, France) both carry a SWIR spectrometer with a point-source telescope (GSD of 50 m) and with a LoD of about 100 kg/h. Data from those micro-satellites are to be correlated with the Copernicus data for reliable assessment.

There is also another weaker absorption line at 1654 nm, often used in active laser-based systems, which will be discussed in Section 2.3.

### 2.2. Elements of Quantitative OGI (q-OGI)

Accurately quantifying methane emissions is critical for industrial reporting, meeting LDAR regulations, and achieving operational excellence. While standard concentration measurement methods give only indirect leak rate estimates through strong plume model assumptions, which can carry large, unacceptable uncertainties, OGI provides a more direct measurement of the actual gas lost to the atmosphere. By analyzing OGI video frames, an approach called quantitative OGI (q-OGI), one can estimate the leak rate (in L/min or g/h) using extracted plume characteristics like shape, contrast, and velocity.

The plume’s velocity field is extracted through well-known optical flow methods applied on consecutive frames, such as block-matching [18] or Lucas-Kanade [19] methods. Recent advances on deep neural network architecture are able to provide high resolution dense flow maps [20], with the emergence of networks specifically trained on OGI video data [21].

Quantification accuracy is mostly a matter of proprietary software from the OGI camera manufacturer. Legacy q-OGI cameras may produce significant errors in real industrial settings, as reported by blind test evaluation with Teledyne-FLIR’s GF320 cooled camera and the QL320 add-on for q-OGI (typical quantification errors from −67% to +200%) [22]. However, recent breakthroughs in data processing, especially those leveraging physics-aware artificial-intelligence methods such as the ‘Smart-OGI’ integrated in SENSIA’s Mileva33 cooled camera [23], enable accurate quantification estimates together with uncertainty. In the recent blind test technology assessment in reference [24], SENSIA’s Smart-OGI emerges as the most accurate quantification technology among participants. Consequently, modern quantification solutions can provide reliable leak rate quantification assessments.

Note that, the difference between cooled and uncooled only impacts sensitivity (notably MDLR) as further explained in Section 4.4. The same quantification methods can be applied to both camera types, assuming sufficient Contrast-to-Noise Ratio.

### 2.3. Other Technologies

Gas detection technologies fall into two categories: in-situ (a.k.a. ‘*sniffers*’) and remote sensors. We will first briefly look at in situ which provide concentration measurement (given in ppm). Two main techniques are identified. The first, NDIR (Non-Dispersive IR), an inexpensive technology that relies on differential quantitative analysis of IR absorption in a gas cell that contains a thermal emitter and a bi-element thermal detector with an active filter and a reference filter. Typical LoD of commercial NDIR sensors are typically below 2 ppm of methane [25]. TDLAS (tunable diode laser absorption spectroscopy) works by modulating a laser’s wavelength (typically via its injection current) around a specific gas absorption line (usually the 1654 nm methane line in the SWIR) within a gas cell. Sensitivities are orders of magnitude below those of NDIR. These techniques provide only local measurements of gas concentration and cannot map the plume’s spatial distribution unless the sensor is moved (e.g., drone’s flight path).

Active remote gas detection technology relies on narrow-band illumination of a gas absorption line and imaging of attenuated back-scattering from the plume. It typically uses a laser tuned to the 1654 nm absorption line in the SWIR, but may use other wavelengths up to the mid-IR. The LiDAR (Light Detection and Ranging) is the standard method for generating a depth map by measuring the time-of-flight of a back-scattered laser pulse, and it can be readily adapted for gas detection by tuning the laser wavelength to a gas’ absorption line. An airbone LiDAR has a LoD of about 1.6 kg/h with calm wind conditions [26]. The laser beam can be wavelength-modulated, much like TDLAS, to perform remote measurement of the gas plume [27]. This approach is sometimes improperly called ‘*laser-OGI*’ when implemented in handheld devices and used the same way as a passive thermal OGI camera, as proposed by Xplorobot (Houston, TX, USA). The remote TDLAS senor does not provide scanning nor 2D imaging data, but is overlayed onto a visible image for contextual information.

Passive thermal OGI is the only time-resolved technology with native imaging capability.

### 2.4. On-Site Technology Deployment

**Figure 2:**
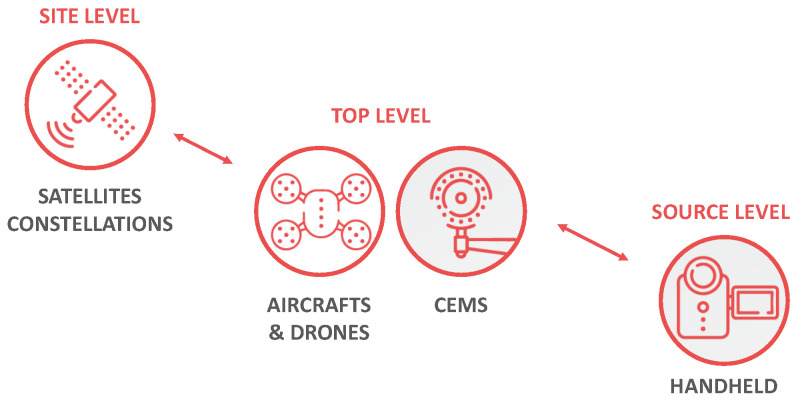
Combination of methane detection technologies across multiple vectors: satellites, drones, fixed and handheld cameras. Double arrows denote the duality of top-down and bottom-up methodologies.

Methane detection and quantification are conducted across multiple scales (see Figure 2), using a combination of periodic measurements from satellites, drones and handheld devices as well as permanent monitoring systems such as a network of in-situ sensors and OGI surveillance cameras. These complementary technologies together provide a comprehensive framework to support timely interventions and reliable regulatory reporting through multimodal confirmation and multiscale reconciliation, as required by OGMP 2.0.

Satellite imagery has a limited sensitivity of a few hundreds of kg/h and can detect super-emitters [28,29] at site-level, bringing global emission reporting and accountability.

Periodic airborne measurement campaigns, such as those conducted with drones or manned aircrafts refine satellite observations with better accuracy and sensitivity. Site-level emission quantification is usually performed with a drone (such as Total Energies’ AUSEA [30]) having a flight-path that samples the gas concentration data in a cross-section perpendicular to the down-wind direction. Drones equipped with OGI cameras are also used to capture plume images from inaccessible areas [31].

CEMS is composed of fixed sensors at the facility level, operating 24/7, provide real-time data on gas concentrations and early detection of leaks, which is particularly useful for high-priority sites where rapid detection is crucial. CEMS can be a network of in situ sensor [32] and/or fixed OGI cameras mounted as surveillance cameras.

Source level detection and localization is usually conducted on a periodic basis by trained contractors (‘*leak hunters*’) equipped with handheld devices such as OGI cameras. The latter is reported to be the better solution for this application [33]. Further inspection of known leaks, from prior OGMP 2.0’s L4 inventory (e.g., detected through CEMS, or top-level surveys), can be carried out to confirm and precisely quantify the leak, and track its evolution before a repair is carried out.

Because of the specific characteristics of cooled and uncooled OGI cameras, we tend to associate cooled cameras preferentially with handheld applications and uncooled cameras with CEMS. In practice, the situation is more nuanced thanks to recent technological advances: the sensitivity of uncooled cameras has improved enough for Type 1 LDAR applications, and the reliability of cryo-cooler has also increased making 24/7 operation viable. For these reasons, an uncooled camera can be handheld, just as a cooled camera can be employed in CEMS. Deciding between cooled or uncooled cameras is mainly determined on the required performances in terms of sensitivity and distance, and how many cameras can be deployed while staying within the available budget envelope. In this manuscript, we will dive into the various characteristics of both cooled and uncooled OGI cameras, especially their performance, to better justify appropriate use cases for each camera type.

## 3. Materials and Methods

We will compare two OGI camera setups: a cooled MWIR camera based on the EOLE MW sensor and an uncooled camera based on the PICO640S BB 7–14. Both sensors are manufactured by Lynred. The two OGI camera setups have similar field of views (FOV), with a VGA (Video Graphics Array) resolution. We compare their key characteristics in Table 1.

EOLE MW’s focal plane array (FPA) features Lynred’s III–V HOT photo-diode technology, operating at a 150 K temperature. It uses a compact split linear cryo-cooler with a total weigh of 368 g and power consumption < 3.4 W [34]. This makes it well suited for battery-powered applications. The MWIR absorbance band of hydrocarbons is targeted with a cold 3.2–3.55 μm band pass filter, with a mean quantum efficiency (QE) of 70% (typical).

PICO640S BB 7–14 is an OGI variant [35] of VOx micro-bolometer array PICO640S [36], which is the state-of-the-art in terms of thermal sensitivity among uncooled thermal sensors. Such a panchromatic (broadband, BB) sensor can target multiple gases, with a ‘hot’ (room temperature) interchangeable optical filter [37,38], such as methane (CH_4_), sulfur-dioxide (SO_2_), nitrous oxide (N_2_O), sulfur hexa-fluoride (HF_6_), and refrigerant gas (R-12, R-134a, R-152a). In our case, we use the band pass filter BBP 7000–8750 nm (manufactured by Spectrogon, Taby, Sweden). Such a filter has an average transmittance of 90% in the 7.0–8.75 μm range, which is well fitted to CH_4_ detection in the LWIR range (see, Figure 1).

**Figure 3:**
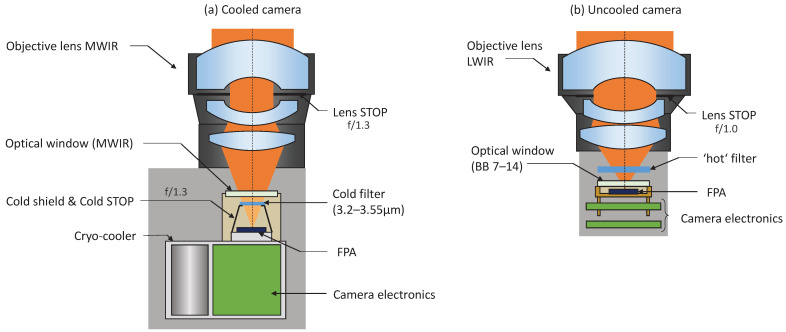
Simplified view of camera assembly: (**a**) cooled, (**b**) uncooled.

The assembly of camera elements is schematized in Figure 3:

NETD (Noise Equivalent Temperature Difference) is a key performance indicator (KPI) for thermal imaging cameras, and will be further discussed in Section 4.2.2. Spectral filtering inherently degrades NETD due to the reduced spectral window. One must not be fooled by NETD degradation, because filtering is crucial for OGI, as it enables a better isolation of the contrast of gas (within its absorption band) from the thermal emission of the background. Using a cold filter is particularly relevant for photo-diodes where the noise is limited by the background photon flux: EOLE MW’s typical NETD is about 22 mK, measured in front of a 27 °C blackbody with a 16 ms exposure time and a 90% lens transmission. Uncooled detectors do not require cold filters, and the panchromatic PICO640S BB 7–14’s measured NETD increases from 19.2 mK up to 85 mK after filtering.

Sensors are fitted with the appropriate lens, which is characterized by its focal length (FL) and ‘*f-number*’ (f/# or N). The FL sets the optical magnification needed for the application: FOV required to image a given scene at a given distance. Horizontal Field of View (HFOV) can be estimated using the paraxial approximation: $HFOV≃2tan^{-1}\left(n_{col}p/2FL\right)$, with $n_{col}$ = 640, and *p* the pixel’s pitch.

The f/# notably characterizes the amount of light the lens is able to collect. The lower sensitivity of the uncooled sensor requires the use of a lens with low f/# (*fast lens*) such as f/1.0, a typical aperture for fast uncooled LWIR lenses. Sensitivity of the uncooled camera can be further improved using even faster lenses down to f/0.8–0.75 usually reserved for high-end systems. Cooled sensors have a cold shield with a given aperture (*cold STOP*): f/1.3 for EOLE MW. Optimal lens aperture should match that of the sensor’s *cold STOP*. Slower lenses are sub-optimal because they can collect less light, and capture more thermal stray-light. Faster lenses have an aperture wasted by the cold shield, thus will not improve sensitivity but only increase weight, cost and complexity of the lens assembly.

In this manuscript, we use the FL 30 mm f/1.1 (reference C-680047-001) with EOLE MW and the FL 35 mm f/1.0 (reference C-65119-04) with PICO640S BB 7–14; both lenses are manufactured by MKS/Ophir optronics.

## 4. Theoretical Principles of Optical Gas Imaging

### 4.1. Radiance Contrast

**Figure 4:**
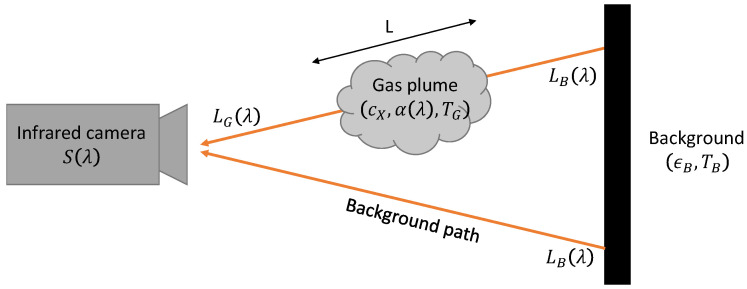
Schematic of a simplified OGI scenario.

An OGI camera is able to visualize a gas plume by thermal contrast with respect to the background. Let us model simply this phenomenon: a thermal IR camera with spectral response S(λ) captures an image of a plume of gas X (e.g., CH_4_), with absorbance α(λ) (in ppm^−1^ · m^−1^), concentration $c_{X}$ (in ppm), temperature $T_{G}$, and pathlength *L* (in m), as schematized in Figure 4. In our model, we neglect the influence of air, an assumption valid for short viewing distances below 2 m.

We model the background as a uniform blackbody of emissivity $\epsilon_{B}≃1.0$ (US-EPA specifies the background emissivity >95%, and the background temperature is given as an ‘apparent temperature’, which implies that the real temperature is adjusted to match the radiance of the equivalent black-body) at the temperature $T_{B}=T_{air}$. Writing $L_{0}\left(\lambda ,T\right)$ as the blackbody spectral radiance (Planck’s law, expressed in W/m^2^/sr/nm) at the temperature *T*, we express the background spectral radiance $L_{B}\left(\lambda \right)$ in the equation below. Note that the reflected radiance from the environment, which takes into account temperature and emissivity is neglected because $(1-\epsilon_{B})\ll 1$ and $T_{env}≃T_{B}$

(1) $$L_{B}\left(\lambda \right)=\epsilon_{B}L_{0}\left(\lambda ,T_{B}\right)+\left(1-\epsilon_{B}\right)\epsilon_{env}L_{0}\left(\lambda ,T_{env}\right)≃L_{0}\left(\lambda ,T_{B}\right)$$

We express $L_{G}\left(\lambda ,c_{X}L\right)$, the spectral radiance along the optical path through the gas, which corresponds to the emission from the background, absorbed by the gas, as well as the emission from the gas itself. The absorbance of the gas (and also its emittance) along L is modeled by a Beer–Lambert law: $e^{-OD(\lambda )}=e^{-\alpha \left(\lambda \right)c_{X}L}$.

(2) $$L_{G}\left(\lambda \right)=e^{-\alpha \left(\lambda \right)c_{X}L}L_{B}\left(\lambda \right)+\left(1-e^{-\alpha \left(\lambda \right)c_{X}L}\right)L_{0}\left(\lambda ,T_{G}\right)$$

**Figure 5:**
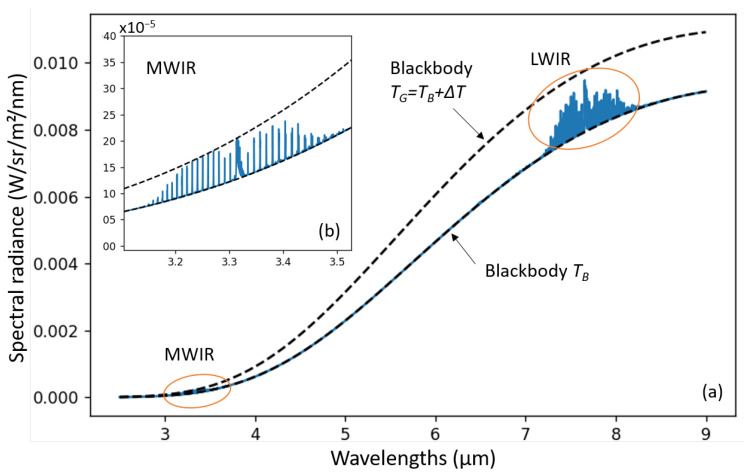
(**a**) Spectral radiance $L_{G}$ along the optical path that goes through a CH_4_ plume, with $c_{X}L$ = 1000 ppm·m, and ΔT=+10 K. (**b**) Zoomed view on the MWIR absorption range.

We plot in Figure 5 the spectral radiance curve $L_{G}\left(\lambda ,c_{X}L\right)$, for a plume of CH_4_, with $c_{X}L$ = 1000 ppm·m; $T_{B}=T_{ref}=296$ K; and $\Delta T=T_{G}-T_{B}=+10$ K.

The radiance curve is bounded by that of the background and that of a black body at gas temperature (dashed curves). In other words, the more the gas temperature differs from the background temperature, the easier it will be to detect.

On this temperature range, thermal contrast $\Delta L_{0}\left(\lambda ,\Delta T\right)=L_{0}\left(\lambda ,T_{G}\right)-L_{B}\left(\lambda \right)$ is stronger in the LWIR band, as seen in Subfigure (b). However, the MWIR band benefits from strong absorbance of CH_4_. Finally, by writing $\tau \left(\lambda ,c_{X}L\right)=1-e^{-\alpha \left(\lambda \right)c_{X}L}$ as the transmission of the gas plume, we can write the radiance contrast between the gas and the background:

(3) $$\Delta L_{G}\left(\lambda ,c_{X}L,\Delta T\right)=\tau \left(\lambda ,c_{X}L\right)\Delta L_{0}\left(\lambda ,\Delta T\right)$$

### 4.2. Camera Sensitivity to Gas’ Concentration-Pathlength

#### 4.2.1. Gas-to-Background Contrast

We integrate the spectral radiance contrast with the thermal camera’s spectral sensitivity in order to estimate the contrast measured by the camera ΔI (expressed in digital levels DL). We assume that the image contrast of the uncooled camera is proportional to the power contrast $\Delta W(c_{X}L)$ (expressed in nW), which is obtained using the following spectral integration:

(4) $$\Delta I\left(c_{X}L,\Delta T\right)\propto \Delta W\left(c_{X}L,\Delta T\right)=A_{p}T_{opt}\Omega \int S\left(\lambda \right)\Delta L_{G}\left(\lambda ,c_{X}L,\Delta T\right)d\lambda$$

With $A_{p}$, the pixels’ sensitive surface area, $T_{opt}$ = 90%, the objective lens average transmission assumed flat on the interest waveband, Ω, the solid angle of the objective lens (or the cold STOP), defined from its f-number N: Ω=2π(1−cos(arcsin(1/2N))).

For a photonic detector, we assume that the image contrast is proportional to the photoelectric current contrast $\Delta \Phi (c_{X}L,\Delta T)$ (expressed in $e^{-}/s$), which is obtained using this alternate expression:

(5) $$\Delta I\left(c_{X}L,\Delta T\right)\propto \Delta \Phi \left(c_{X}L,\Delta T\right)=A_{p}T_{opt}\Omega \int \frac{\lambda }{hc}QE\left(\lambda \right)\Delta L_{G}\left(\lambda ,c_{X}L,\Delta T\right)d\lambda$$

With h the Planck’s constant and c the speed of light: hc/λ is the energy of a single photon with the wavelength λ. We can rewrite image contrast for both sensor types, as a function of temperature difference and concentration length:

(6) $$\Delta I\left(c_{X}L,\Delta T\right)=G\int \tilde{S}\left(\lambda \right)\Delta L_{G}\left(\lambda ,c_{X}L,\Delta T\right)d\lambda$$

With $\tilde{S}\left(\lambda \right)$ being the normalized spectral response of the sensor (arbitrary unit). For a thermal sensor, we define $\tilde{S}\left(\lambda \right)\propto S\left(\lambda \right)$; for a photonic sensor: $\tilde{S}\left(\lambda \right)\propto \left(\lambda /hc\right)\cdot QE\left(\lambda \right)$. G is the camera gain factor, which expresses the conversion of radiance into digital levels (DL), and is expressed either in (DL/W/m^2^/sr) or in (DL/ph/s/m^2^/sr) depending on the sensor type.

#### 4.2.2. Contrast-to-Noise Ratio (CNR) from NETD

The responsivity R (expressed in DL/K) at a reference temperature $T_{ref}$ quantifies the camera’s signal variation due to a scene temperature variation; we expressed as the scene temperature partial derivative of the camera signal.

(7) $$R\left(T_{ref}\right)=\frac{\partial I}{\partial T}=G\frac{\partial }{\partial T}\left[\int \tilde{S}\left(\lambda \right)L_{0}\left(\lambda ,T\right)d\lambda \right]\left(T_{ref}\right)$$

The responsivity is measured in a factory using hot and cold blackbody references $\left(T_{cold},T_{hot}\right)$ = (20 °C, 35 °C). This metric exhibits non-linearities depending on the set of reference temperatures, and the measurement is accurate only within the temperature range in the vicinity of those reference blackbody temperatures.

(8) $$R\left(T_{ref}\right)≃\frac{I\left(T_{hot}\right)-I\left(T_{cold}\right)}{T_{hot}-T_{cold}}$$

NETD (expressed in mK) is the standard noise metric for thermal cameras [39]; it converts the camera noise σ(T) (signal’s temporal standard deviation, expressed in DL) into temperature units.

(9) $$NETD\left(T_{ref}\right)=\frac{\sigma (T_{ref})}{R(T_{ref})}$$

Note that we consider only temporal noise and not residual fixed pattern noise (RFPN). Indeed, we assume appropriate non-uniformity correction (NUC) where RFPN is small compared with temporal noise. Moreover, processing such a differential imaging suppresses background and thus RFPN, as later described in Section 6.2.

NETD, as a noise estimator, is accurate only when the noise does not vary much when the scene temperature differs from that of the reference. This is the case for uncooled micro-bolometers, where the dominant noise is Johnson’s (noise from the sensor’s thermo-resistance).

The Contrast-to-Noise Ratio (CNR) at a given ΔT is calculated using the following expression:

(10) $$CNR_{\Delta T}\left(c_{X}L\right)=\frac{\Delta I(c_{X}L,\Delta T)}{\sigma (T_{ref})}=\frac{G\int \tilde{S}\left(\lambda \right)\Delta L_{G}\left(\lambda ,c_{X}L,\Delta T\right)d\lambda }{NETD\left(T_{ref}\right)R\left(T_{ref}\right)}$$

This estimation of CNR requires an accurate value of NETD, as such a measurement is made under similar conditions to that of final use in terms of camera and scene temperature ranges.

#### 4.2.3. CNR from BLIP Model

A Background Limited Infrared Photodetector (BLIP) is a photodetector model where one considers that the noise is dominated by the shot-noise from the background, compared with other noise contributors (such as $\sqrt{kTC}$ and readout noise). This model is valid for photonic detectors with proper exposure time and well fill (≃50%). The background shot-noise is the square root of the collected background photo-electrons during the exposure time $t_{exp}$. Note that the absolute value of the quantum efficiency must be known. For EOLE MW, average QE is around 70%.

(11) $$\sigma_{BLIP}^{2}\left(T_{B}\right)=t_{exp}A_{p}T_{opt}\Omega \int \frac{\lambda }{hc}QE\left(\lambda \right)L_{B}\left(\lambda \right)d\lambda$$

CNR for a BLIP model becomes:

(12) $$CNR_{\Delta T}\left(c_{X}L\right)=\sqrt{\frac{t_{exp}A_{p}T_{opt}\Omega }{\int \left(\lambda /hc\right)QE\left(\lambda \right)L_{B}\left(\lambda \right)d\lambda }}\int \frac{\lambda }{hc}QE\left(\lambda \right)\Delta L_{G}\left(\lambda ,c_{X}L,\Delta T\right)d\lambda$$

#### 4.2.4. Noise Equivalent Concentration Length

NECL (Noise Equivalent Concentration Length, expressed in ppm·m) is a metric that quantifies a camera sensitivity to a gas’ concentration-pathlength, proposed by Teledyne-FLIR [40]. NECL is defined with a thermal contrast ΔT = 10 K at a 1 m viewing distance as the following.

(13) $$CNR_{10K}\left(NECL\right)=1$$

**Figure 6:**
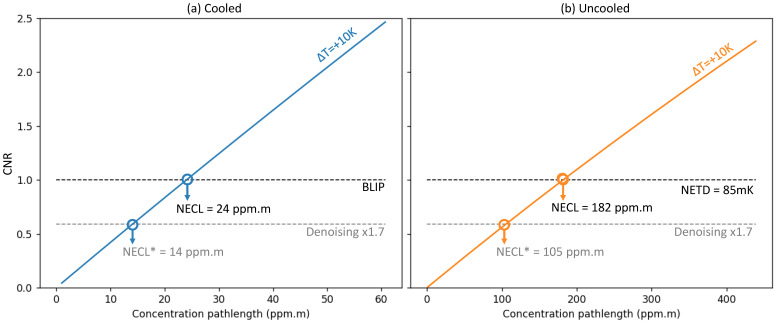
Gas-to-background Contrast-to-Noise Ratio, at ΔT=10 K: (**a**) cooled, (**b**) uncooled.

We plot in Figure 6 the $CNR_{\Delta T}\left(c_{X}L\right)$ curves for both cameras. For the uncooled camera, noise is computed with NETD = 85 mK (typical), which accounts for the filter spectral transmittance at ambient temperature. For the cooled camera, we use the BLIP model, which gives the same results as using the measured NETD = 22 mK (with optics). Native NECL is the concentration path-length value obtained when the $CNR_{10K}$ curve crosses the black dotted line (CNR = 1), which gives a native NECL of 182 ppm·m for the uncooled camera, and 24 ppm·m for the cooled camera.

OGI cameras have NECL usually given with noise reduction post-processing (‘*denoising*’) disclosed in data-sheets. Denoising consists in reducing the noise, while preserving the signal through careful design and parameter setting. We will propose an efficient noise reduction algorithm in Section 6.2, where empirical studies showed improvement in measured CNR by a factor ranging from 1.64 to 1.78 depending on the sequence and algorithm settings. We use a reasonable denoising factor of ×1.7: this gives us a post-processed NECL* of 105 ppm·m for the uncooled camera, and 14 ppm·m for the cooled camera. The * is used to indicate denoising, in order to avoid confusion with native sensor performances.

This theoretical sensitivity study suggests that the cooled camera (EOLE MW) is ×7.5 more sensitive to CH_4_ than the uncooled one (PICO640S BB 7–14). This value is a result of multiple parameters such as NETD (better for cooled), spectral features intensity (better in MWIR) and thermal contrast (better in LWIR). NECL measurement is not trivial, and requires the use of windowed gas cells of known concentration to be viewed with a temperature controlled background [37,38,40].

**Figure 7:**
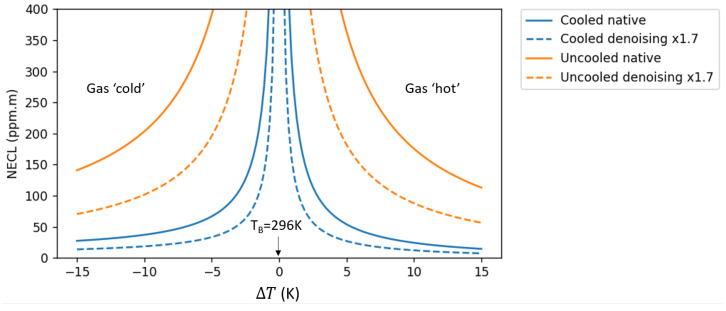
NECL and NECL* vs. temperature difference ΔT for both the cooled and uncooled cameras.

NECL is given by default at a gas-to-background temperature difference ΔT=+10 K. The gas plume gets harder to detect when the gas temperature gets close to the background temperature (ΔT≃0 K). This case almost never happens in practice, notably because uniform backgrounds do not exist in real settings, and also because the gas is usually not at ambient temperature mainly due to pressure conditions inside the gas pipes. We plot in Figure 7 the NECL and NECL* as a function of temperature difference ΔT. Note that there is asymmetry of CNR with respect to the temperature difference polarity, with better performances when the gas is hotter than the background. There is a greater sensitivity when the gas is seen with a cold sky background.

### 4.3. Gas Plume Model Leak Rate

MDLR (given in g/h) is another KPI for OGI camera, which is explicitly disclosed in both USA and EU rules (<17 g/h Type 1 LDAR). It means that the OGI camera must render an image in which a camera operator can detect a gas leak. MDLR is a metric that can be highly biased by both the operator’s visual acuity and the ‘controlled’ laboratory conditions such as wind-speed intensity (assumed low) and orientation, exit velocity and outlet diameter, etc., which are not properly defined in the regulations.

A leak plume can be detected if an operator can resolve the plume with sufficient CNR. To assess this, one needs to model such a plume in order to generate a three-dimensional concentration map that models the dispersion of the gas. This concentration map is projected into a concentration path-length (CL) sampling array, representative of a noise-free camera image, which is then compared with the camera’s NECL* at the given ΔT.

We use the simple Gaussian dispersion plume model, as described by M. R. Beychok [41]. Such a model describes experimental data (averaged OGI images) from controlled leaks, as shown in Reference [19]. However, it generates smooth and continuous plumes, and thus cannot generate video sequences that carry the displacement of in-homogeneous ‘swirls’ of higher and lower concentrations.

#### 4.3.1. Gaussian Dispersion Plume Model

We consider the Gaussian plume, defined by its concentration C(x,y,z) as detailed in Appendix A.2. The OGI camera records an image of said plume with an optical axis parallel to the y-crosswind direction. To simplify the problem, we ignore perspective effects so that we can simply integrate the optical pathlengh in the y-direction. Expression of the concentration-pathlength CL(x,z) in object space (given in ppm·m) can be thus simplified:

(14) $$CL\left(x,z\right)=\int C\left(x,y,z\right)dy=\frac{Q}{u}\frac{g(x,z)}{\sigma_{z}\left(x\right)\sqrt{2\pi }}$$

*Q* is the leak rate given in (g/s). Effective wind-speed *u* (in m/s) is projected in the transverse optical plane.

**Figure 8:**
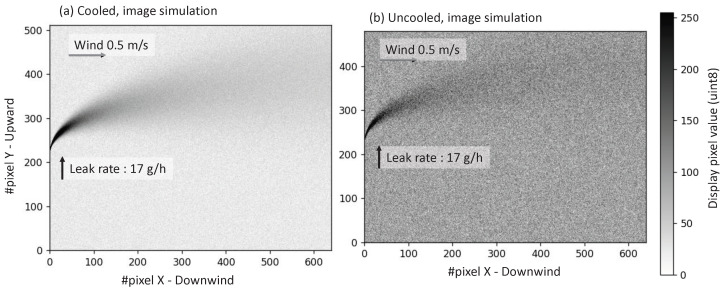
Image simulations of the same methane plume (17 g/h leak rate) using both the (**a**) cooled and (**b**) uncooled OGI cameras.

Conversion from object space (x, z in m) to image space (i, j in pixels) is done using the optical magnification M (in pix/m), assuming no optical distortions: M=FL/p·d. We will use the following simulation parameters for our Gaussian plume: viewing distance d = 2 m, $h_{0}$ = 0.2 m, horizontal wind-speed 0.5 m/s, ΔT = +5 K. The same plume is seen with both OGI cameras with similar HFOV, with the parameters previously summarized in Table 1. We show in Figure 8 a comparative image rendering of the same plume of methane (Q = 17 g/h leak rate) using both cooled and uncooled OGI cameras. We add Gaussian noise to the CL images with amplitude NECL* (values taken at ΔT = 5 K, with the ×1.7 noise reduction enabled): 31.6 ppm. and 223.6 ppm·m for the cooled and uncooled cameras respectively. Those images are then rescaled to the 8-bit display dynamic. This simulation is representative of the laboratory conditions of the EU and US regulations.

The cooled camera shows a clear image of the 17 g/h methane leak. As expected, the uncooled image has significantly more noise, but any operator should properly resolve the plume in those conditions. It suggests that both the cooled and uncooled cameras would be compliant with EU’s Type 1 LDAR (<17 g/h, ΔT unspecified) and US regulations (<19 g/h, ΔT=5 K).

#### 4.3.2. MDLR Simulation

The MDLR criterion is unclear and based on the camera operator acuity. We propose to define $N_{pix}$ as the number of pixels where $CL\left(i,j\right)>NECL^{*}$. Reference [19] suggests to set the criterion at $N_{pix}$ = 400 pix, which corresponds to an equivalent square of width of 20 pix. However, we argue that a 9 × 5 pix criterion is still reasonable, especially if the operator is assisted visually with proper image representation such as plume specific colorization (see Section 6).

**Figure 9:**
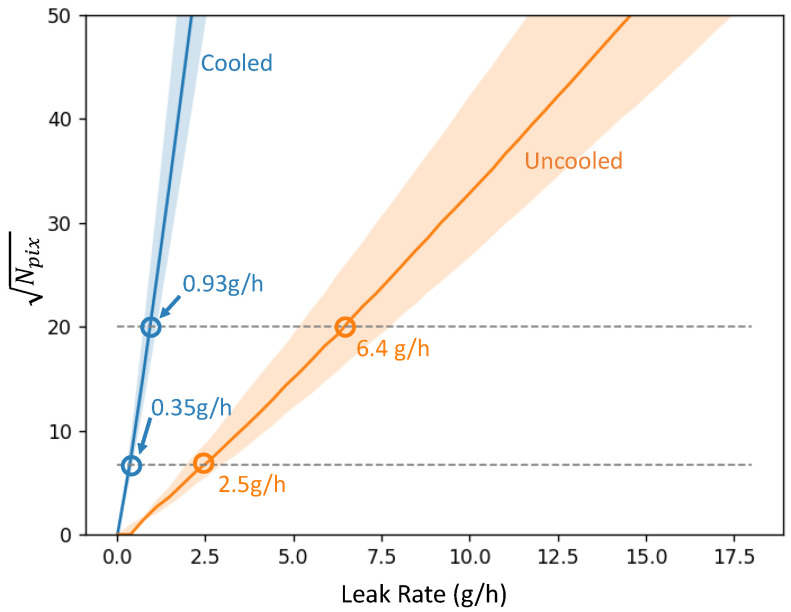
Square-equivalent width of the detected plume as a function of the leak rate, for both cameras. Wind is u=0.5±0.1 m/s.

We plot in Figure 9, the square-equivalent width $\sqrt{N_{pix}}$ of the detected plume for both cameras. MDRL* values are obtained when $\sqrt{N_{pix}}$ reaches a given threshold. Assuming good laboratory conditions, the cooled cameras would even be compliant with Type 2 LDAR (<1 g/h, ΔT unspecified).

Absolute values of MDLR* are highly biased due to the conditions, notably winds and viewing conditions. Indeed, wind speed dramatically changes the dilution of pollutant (colored areas in Figure 9 correspond to ±0.1 m/s in wind-speed), and thus the projected concentration-length; the distance and FL modify the optical magnification, and thus the sampling quality at the vicinity of the plume exit.

Note that real turbulent plumes have swirls of higher and lower concentration. Our Gaussian model is only representative of time-averaged images. It gives, however, good insights into the dependencies of MDLR* which are in good accordance with the extensive empirical study found in Reference [42].

- MDLR* is proportional to the concentration pathlength detection limit (NECL* at given ΔT). Better MDLR* is expected when the thermal contrast is high, notably with a cold sky background.
- Distance does not influence the result at first order, but optical magnification and sampling does.
- Pixel count is dependent on the spatial resolution of the plume: high definition camera with narrow field of view improves MDLR*.

### 4.4. Summary of KPIs

To sum up, we assessed KPIs for OGI cameras: NETD*, NECL* and MDLR*, which can provide insight whether or not a leak can be detected. These metrics represent the LoD for various quantities under given operating conditions. The * denotes the denoising step, allowing for lower LoDs through post-processing.

- NETD corresponds to the camera noise level, converted to temperature units (mK). This sensitivity metric represents the minimal difference of temperature one can detect and is notably a combination of the sensor’s sensitivity and the lens’ aperture. NETD is not specific to OGI, but generic to thermal cameras. Low NETD does not mean good OGI performances! Indeed, a better spectral specificity (finer bandwidth) degrades NETD but improves the gas contrast with respect to the background.
- NECL is better suited for OGI: it represents the camera’s LoD to a concentration-pathlengths (in ppm·m) for a given gas. This metric is defined at 1 m distance, with a ΔT of 10 K. It sums up multiple parameters such as in-band gas opacity, apparent thermal contrast, and NETD.
- MDLR is the leak rate LoD (in g/h) for a given gas. This multi-parametric KPI is defined as in the regulations: can an operator detect a leak through the camera display, at a 2 m distance and ΔT of 5 K? MDLR depends on the camera resolution (≃number of pixels), optical magnification (viewing distance and HFOV) and display mode. Although biased by the human factor, this metric is appropriate for LDAR applications where the operator’s sense of observation does matter in the end.

We summarize in Table 2 those KPIs for both cooled and uncooled, which where obtained through simulation:

We recall that theoretical predictions of NECL and MDRL imply controlled laboratory conditions with uniform background, short distance (atmospheric effects neglected) and calm wind. Detection of a gas leak in real field conditions will deviate from our model (see Section 6).

Manufacturers specify an OGI camera’s MDLR through controlled laboratory evaluation (and not simulated), which includes human influence. We should confirm that the theoretical analysis predicts MDLR values that match the magnitude observed in those laboratory measurements, while keeping in mind the highly biased nature of the MDLR assessment.

## 5. Laboratory Leak Rate Evaluation

**Figure 10:**
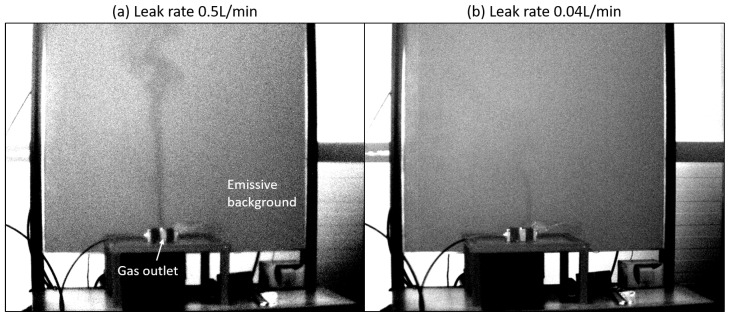
Controlled leaks of pure methane in laboratory settings. Leak rates: (**a**) 0.5 L/min and (**b**) 0.04 L/min.

MDRL evaluation is performed in laboratory settings, in conditions close to that described in regulations: with a controlled methane leak of known rate, where the plume in seen by contrast to a uniform background at ambient temperature. There is no precise way to control ΔT, but it should be in the order of magnitude of 5 K, thanks to the gas’ expansion from the pipe pressure to ambient pressure. To our knowledge, this is how OGI cameras are tested for regulatory compliance. Laboratory trials were conducted using the cooled camera: EOLE with a suboptimal FL 25 mm f/2 lens (Infra-MW252.0 from Wavelength Opto-Electronic Singapore) at 4 m distance (viewing distance is increased from the 2 m specified in regulations for security reasons as our reasearch material is not qualified for explosive atmospheres. MDRL is expected to be better at this closer distance). Note that better performances are expected with a f/1.3 lens, properly adapted to the sensor’s cold stop aperture. Snapshots from the camera video stream are shown in Figure 10 with leak rates decreasing from 0.5 L/min down to 0.04 L/min.

The image is simply processed with a non-uniformity correction (NUC), and linear tone-mapping is displayed as a grayscale image without noise reduction post-processing. In accordance with the theoretical study, the Contrast to-Noise Ratio decreases with the leak-rate, which makes the gas leak harder to detect (CL closer to the camera’s NECL), especially with the 0.04 L/min leak rate. Note that, in this case, the gas appears darker than the background (at 22 °C); it means that the gas is colder than the background (ΔT < 0) due to expansion as it escapes through the leak. We verify that real gas plumes have patches, unlike our smooth Gaussian model.

In practice leak rates are given in volume flows (L/min) as read by the flow meter. Because the regulations require flows to be given as a mass flow rate (in g/h), we convert L/min to g/h by the following factor, that depends on the pressure and temperature:

(15) $$M_{g}\frac{p}{RT}\frac{60}{1000};[g\cdot h^{1}\cdot L^{-1}\cdot \min]$$

Assuming $M_{g}=16.0425$ g/mol the molar mass of pure methane, p = 101,325 Pa (1 atm) the pressure, T = 296 K the temperature and R the perfect gas constant; we have a conversion factor of 39.63 g·h^−1^ · L^−1^ · min. Thus the 0.5 L/min volume flow rate in Subfigure (a) corresponds to 19.82 g/h which is about the leak rate imposed by the regulations (US-EPA, and EU’s Type 1 LDAR); the 0.04 L/min in Subfigure (b) corresponds to 1.59 g/h which is close to EU’s Type 2 LDAR. This trial tends to confirm the MDLR orders of magnitude obtained in the simulation (Section 4.3.2).

MDLR is measured in very favorable laboratory settings: short distance, calm wind and uniform background. Going from the laboratory to the field will come with new challenges, as we will see in the next section.

## 6. Image and Video Processing with Complex Backgrounds

An OGI camera, as defined in the regulations, must deliver a gas leak image that the camera operator can readily interpret. Therefore, the way the image is presented is critical: the display should show the plume as clearly as possible while imposing minimal cognitive load on the operator. This section outlines the key image and video processing steps such as denoising, separation of the gas plume from the background, and the perception-enhanced image rendering, which are inspired state-of-the art commercial OGI cameras.

Video-processing algorithms should be embeddable, meaning they must rely solely on simple operations that can run on low-power image signal processors (ISPs) [43], generally 1 W or less, enabling their use in battery-operated portable devices.

### 6.1. Classical Representation: ‘White-Hot’

A thermal camera image is usually displayed in grayscale: the so-called ‘*white-hot*’ representation, a display mode favored by the military or experienced thermal camera operators. The raw 16 bit pixel values (with classical NUC) encode scene radiance; before display they are reduced to 8 bits by applying a linear tone-mapping that discards a low percentile portion of the pixel data (hot and cold saturation).

**Figure 11:**
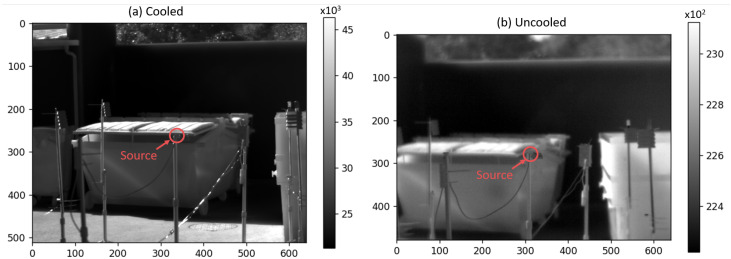
Raw images of the (**a**) cooled and (**b**) uncooled cameras with the ‘white-hot’ representation. Greyscale colormap indicates the input 16-bit pixel values.

Let us take as an example a plume seen with both the cooled and uncooled camera in the same configuration as described in Table 1. The plume originates from a controlled pure-methane leak at a flow rate of 20 L/min (about 0.8 kg/h), and is observed by the cameras from a distance of 6 m as shown in Figure 11. The gas release was made outdoors, at the eLichens facilities (Grenoble, France) with clear weather, 19.7 °C ± 0.2 °C ambient temperature, 58%±1% relative humidity, and 1.8 m/s ± 1.1 m/s horizontal wind speed.

The source is marked by a red circle at the end of the pipe outlet. Bin covers are a few meters in the background and appear blurry, especially in the uncooled image (Subfigure (b)) due to its shorter depth of field.

**Figure A3:**
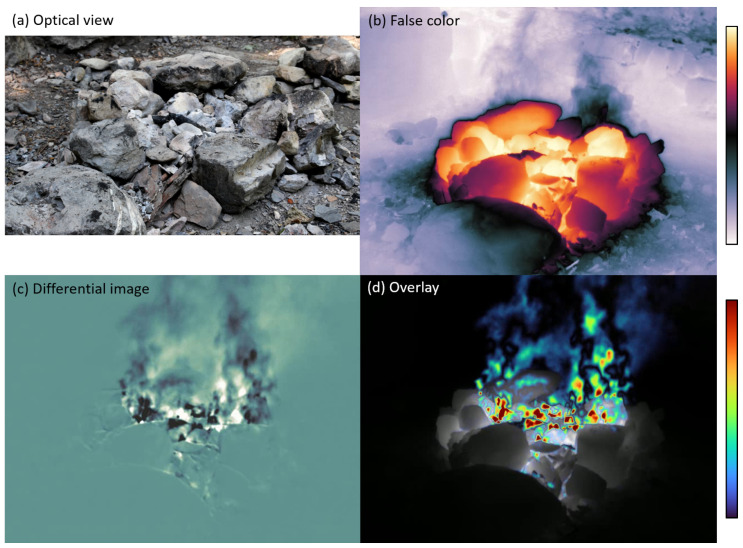
(**a**) Optical view of a natural gas source, (**b**) Snapshot view with the uncooled camera (colorized: *Lifeinred^TM^ contrast*), (**c**) differential and (**d**) ‘*overlay*’ display mode.

In this example, the thermal amplitude of the backgrounds overwhelms that of the gas, making the plume almost invisible, especially on a still image. Grayscale representation does not provide sufficient perceived luminance contrast to properly perceive the plume. A false color representation with high perceptual dynamics, such as one with a high dynamic of color luminance (see Figure A3), or even a rainbow palette, could mitigate this problem, but at the cost of increased cognitive load, to a possibly unacceptable level [44].

### 6.2. Background Subtraction

The plume is extracted from the background based on the assumption that the latter is static and the gas plume consists of swirls that move from one frame to the next. The background image is estimated by applying a temporal filter to the raw image stream. We use an infinite-support exponential filter that blends the current frame $I_{t}$ with the previous filtered frame $I_{t-1}^{*}$, using a fixed weight $p_{0}$. This operation requires the ISP to have only a single frame ($I_{t-1}^{*}$) stored in memory.

(16) $$I_{t}^{*}=p_{0}I_{t}+\left(1-p_{0}\right)I_{t-1}^{*}$$

Initialized at $I_{0}^{*}=I_{0}$. The filter’s characteristic time corresponds to the duration of $1/p_{0}$ frames. We set $p_{0}$ = 1/15 as a fixed weight. This is an empirical value (tested on both the cooled and uncooled configurations) which efficiently reduces the noise. This corresponds to a response time (memory/lag) of 15 frames, or 0.5 s at a 30 Hz framerate and 0.25 s at a 60 Hz framerate. Further reducing the weight does not drastically improve the noise, but increases the filter’s response time. This filtered image carries the information of the static background (with temporal noise attenuated by the filter), and a smoothed plume that no longer retains the details of the moving swirls.

The differential image $\delta I_{t}$ is obtained by subtracting the previous filtered image $I_{t-1}^{*}$, assimilated as the background.

(17) $$\delta I_{t}=I_{t}-I_{t-1}^{*}$$

It carries the information about the motion of the plume’s swirls, but also about other scene movements (elements blown by the wind such as foliage, flying insects, camera jitters, etc.). The noise level is comparable to the original sensor noise, so we apply the following spatio-temporal denoising stage:

(18) $$\delta I_{t}^{*}=K⊛\left(p\delta I_{t}+\left(1-p\right)\delta I_{t-1}^{*}\right)$$

Initialized at $\delta I_{0}^{*}=K⊛\delta I_{0}$. Where K is a Gaussian kernel, and ⊛ the convolution operator. We justify applying a spatial filter on the premise that the gas plume is inherently diffuse and lacks distinct, sharp boundaries (i.e., it contains little or no high-frequency information).

Here, the weight *p* is not fixed, but a pixel-wise function of image difference $\Delta I=\delta I_{t}-\delta I_{t-1}^{*}$, defined as an activation function based on a noise estimation σ. The definition of p(ΔI) is not explicitly disclosed, but behaves as follows: when |ΔI|≪σ, we assume no motion so we accumulate associated pixel values with a weight $p_{0}$; when instead |ΔI|≫σ, we consider this motion so we update the pixel values.

Noise estimation σ is derived from the camera calibration, by taking the temporal deviation of a uniform reference scene from a prior factory calibration or from a mechanical shutter. This solution is the better choice for the uncooled sensor, where noise is independent of the scene radiance. The noise from the cooled sensor is assumed photon-limited, as explained in Section 4.2.3, and thus estimated from the pixel value of the background image: $\sigma^{2}\propto I_{t-1}^{*}$.

**Figure 12:**
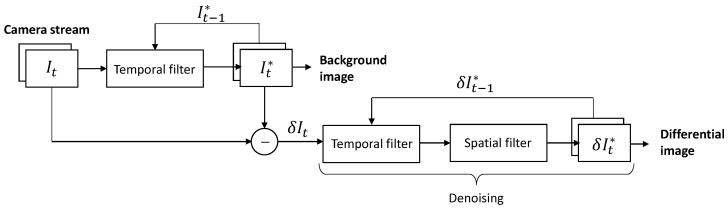
Functional block diagram of denoised differential image pipeline.

We summarize the processing pipeline for the denoised differential image through the functional block diagram in Figure 12.

**Figure 13:**
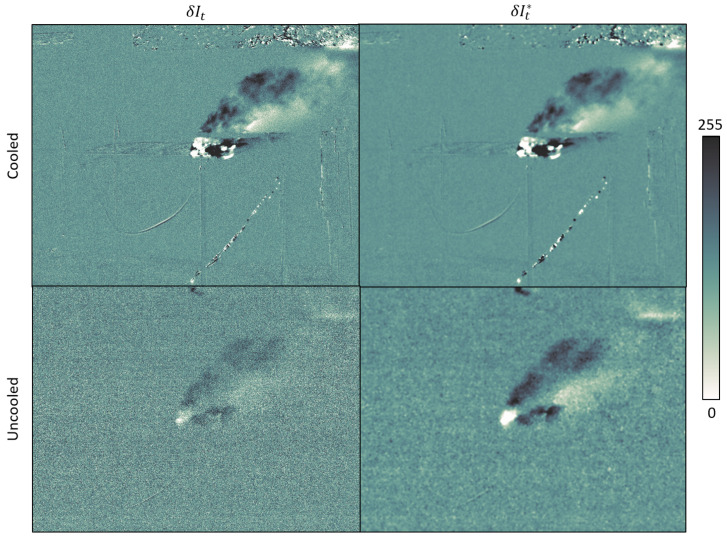
Differential images of the same plume with both the cooled (**top**) and uncooled (**bottom**) cameras before (**left**) and after (**right**) denoising.

Denoising parameters such as filter memory time, activation function threshold and sharpness, spatial filter kernel size, etc., must be optimized empirically depending on the camera type, plume thermal contrast and user preference. We illustrate the effect of the spatio-temporal filter in Figure 13. Due to the higher sensitivity of the cooled camera, we use softer denoising parameters than with the uncooled camera. It allows to damp the noise while preserving details on the swirls, without perceptible smoothing.

The differential images are rendered with a standard rescaler, meaning that the 8-bit image dynamic is mapped linearly from a given factor of the standard deviation of the original gas image. It is then colorized with Lynred’s *Lifeinred^TM^ serenity* color palette. Such a palette is a perceptually uniform [45] colormap, specially designed to reduce cognitive fatigue; thus, adapted to those noisy, unnatural gas plume video sequences. This representation resembles the ‘*High Sensitivity Mode (HSM)*’ used in state-of-the-art OGI cameras [46,47].

This display mode is effective for visualizing the gas plume even when its contrast is low relative to the scene contrast. It is the preferred mode for detecting small gas leaks. Its main drawback is the loss of contextual information, which makes locating the source harder: this drawback will be addressed in Section 6.4. It should be noted that the amplitude of swirls depends on the background, with a contrast inversion when the background is hotter or colder than the gas temperature, as expected from Equation (3).

### 6.3. Contrast Normalization

**Figure 14:**
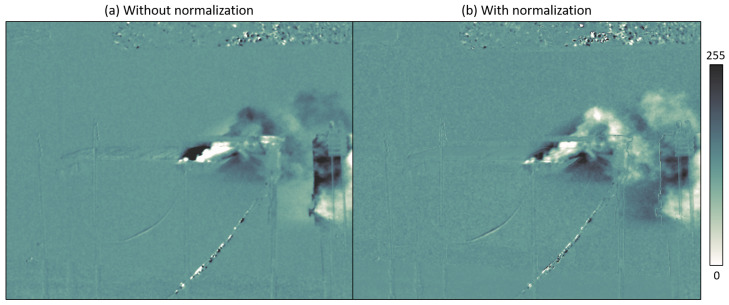
Effect of the contrast normalization step.

The normalization step allows correcting contrast variations resulting from the thermal contrast between the gas and background; more specifically, contrast inversions. We give in Figure 14 an example of normalization on a frame from the cooled camera, where the plume covers a highly contrasted background, with a cold wall and plastic bin covers heated by the sun. This normalization step amplifies the gas contrast locally, resulting in a (controlled) amplification of noise on certain regions of the frame.

Let us describe the necessary operations for such a rendering. Those operation are independent from one frame to another, thus we will simplify the notations by discarding the temporal index t. The normalized gas image ${\tilde{I}}_{G}$ is calculated as such:

(19) $${\tilde{I}}_{G}=\frac{\delta I^{*}-\langle \delta I^{*}\rangle }{Norm}$$

The differential image is centered by its mean value, and divided by the Norm image, which represents the thermal contrast between the background (estimated $I_{t-1}^{*}$) and $v_{ref}$. This latter scalar value (in DL) expresses the radiance value where the gas temperature equals that of the background, which corresponds to the contrast inversion. Estimation of $v_{ref}$ can be derived from an external ambient temperature reading or from the image of a closed mechanical shutter.

(20) $$Norm=\left\{\begin{array}{ccc} \left(I_{t-1}^{*}-v_{ref}\right)/C_{0} & ; & |I_{t-1}^{*}-v_{ref}|>C_{0}\\ sign(I_{t-1}^{*}-v_{ref}) & ; & |I_{t-1}^{*}-v_{ref}|\leq C_{0} \end{array}\right.$$

We limit division by a small number (when $I_{t-1}^{*}≃v_{ref}$), which would result in unacceptable amplification of noise, by clipping small contrast values below $C_{0}$. This damps the normalization correction at the transition between cold and hot objects of the background.

### 6.4. Overlay Mode

**Figure 15:**
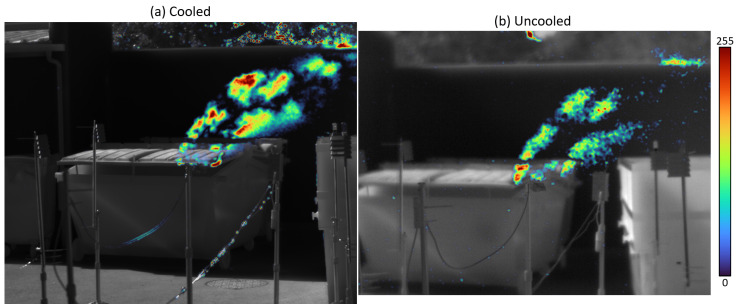
Overlay display mode with the (**a**) cooled and (**b**) uncooled cameras.

The *overlay* mode, inspired from the state-of-the-art [48], consists in superimposing the colorized plume (normalized or differential) on the *white-hot* image with a transparency mask. It simplifies interpretation of the video, and further reduces the cognitive load compared with the differential mode (*High-Sensitivity Mode*) as in Figure 13. An example for the *overlay* representation is given in Figure 15.

Let us describe the necessary operations to render such an image. We take the absolute value of differential or gas normalized image $|{\tilde{I}}_{G}|$, rescaled on an 8-bit display dynamic. Taking the absolute value allows the positive and negative values of the swirls to be displayed in the same way. We clip the low values as a factor of the noise estimation σ that are considered as noise and filtered out.

The 8-bit gas image is colorized with Matplotlib’s (version 3.10.9) ‘*turbo*’ colormap, a color LUT (Look-Up Table) that associates each of the 256 input values to an RGB8 triplet: $I_{G,RGB8}=colormap\left(I_{G,u8}\right)$. It offers a neutral luminance range with a vivid rainbow-like hue progression. It overlays nicely on dark, light, or textured backgrounds, and its visual character is fitting for depicting gas.

The opacity mask, referred to as the Alpha channel α (in float32 format) is derived from $I_{G,u8}$ and is designed to reduce visibility of low values α≃0, while displaying high contrast swirls will full opacity α=1.

Finally, we overlay the colorized gas image $I_{G,RGB8}$ on the *white-hot* image $I_{G,RGB8}$ (both in RGB8 format) using the Alpha channel α. Alternatively, the *white-hot* image can be replaced with a visible image (if available) or a visible-IR fused image.

(21) $$I_{overlay}=\alpha I_{G,RGB8}+\left(1-\alpha \right)I_{BW}$$

**Figure 16:**
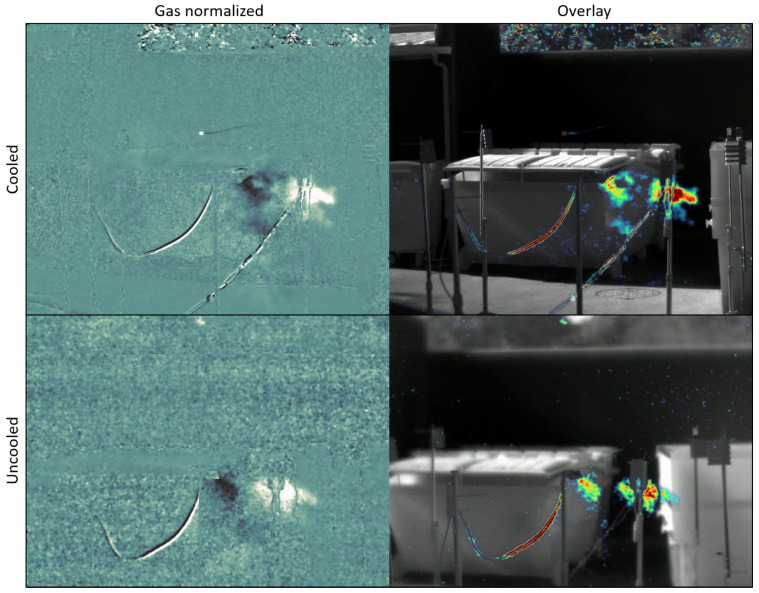
Example of a methane leak at 5 L/min with the cooled (**top**) and uncooled (**bottom**) cameras. Normalized gas display (**left**) and overlay (**right**).

We have shown a single methane leak at 20 L/min (0.8 kg/h). Let us show a fainter leak in Figure 16, at 5 L/min (0.2 kg/h) seen in the same previous conditions and displayed with the processing described above. Lower leak rates result in a plume of reduced contrast, smaller size and slower swirls.

Note that these processing modes are only designed for the purpose of efficient visualization (in normalized 8-bit pixel format), and are unable to provide quantitative data (ppm·m or g/h). q-OGI, as introduced in Section 2.2 is a whole new topic, and is not further discussed in this article.

Other image examples are given in Appendix B.

## 7. Conclusions

We discussed why OGI is valuable for reducing greenhouse-gas emissions, notably methane (CH_4_), in the oil and gas industry. This can be performed at various scales, ranging from satellite-based monitoring to fine, source-level detection, and through continuous emission monitoring systems (CEMS). We detailed the infrared (IR) image sensors suitable for such applications, covering the characteristics of a two camera setup based on two sensors from Lynred: EOLE MW, a high-performance cooled sensor-tailored hydrocarbon detection, with a narrow-band sensitivity in the MWIR (3.2–3.55 μm); and PICO640S BB 7–14, an uncooled sensor filtered in the LWIR’s specific CH_4_ band (7.2–8.3 μm), which offers an excellent performance-to-cost trade-off.

We presented the opto-electronic design principles of passive thermal IR OGI cameras, both cooled and uncooled, with a theoretical optical-radiometric model of plume contrast and detection sensitivity. This allowed us to simulate the performance of OGI cameras applied to CH_4_, and discuss KPIs such as NETD, NECL, and MDLR together with parameters that influence them, such as ΔT, spectral absorbance, camera resolution, optical magnification, or video post-processing. We found that the cooled camera offers a sensitivity advantage of about ×7.5 over the uncooled camera in terms of NECL. Both camera types are compatible with handheld source-level application, and comply with regulation from US-EPA’s QuadOs and EU’s Type 1 LDAR, for being capable of detecting methane plumes below 17 g/h leak rates under laboratory conditions (simulated and experimentally verified) when the background is uniform with a ΔT = 5 K. Indeed, MDLR was calculated as 0.35 g/h and 2.5 g/h for the cooled and uncooled cameras respectively, with assumptions on the visual acuity of the camera operator. Because of its higher sensitivity, the cooled camera is the preferred choice for tasks that demand detecting very subtle leaks, such as Type 2 LDAR (<1 g/h), or airborne (drone) surveys.

Both cameras can reliably operate 24/7, making them suitable for CEMS surveillance cameras. On the one hand, the less sensitive, yet cost-effective, uncooled camera can be distributed across an industrial site at short range. On the other hand, a single cooled CEMS camera can monitor a wider area at longer range (typically 100–200 m).

Finally, we showcased field examples taken outdoors under windy conditions and cluttered scenery where the gas plume was barely perceptible in conventional thermal images. To address this, we described video-processing approaches tailored for OGI: a differential-imaging mode that boosts plume contrast and removes background; and an overlay mode that reduces the user’s cognitive load by layering a colorized plume onto a grayscale contextual image. Effective OGI visualization is not only essential for LDAR when a human operator ultimately finds the leak, but also for clearer reporting.

Gas detection technologies such as NDIR or TDLAS provide data on the gas concentration (in ppm), and sometimes with (incomplete) spatial resolution. Leak rate estimation is done indirectly with strong assumptions, resulting in somehow unreliable data of the true amount of gas lost to the atmosphere. OGI is the only technology able to resolve a gas plume and its swirls, both spatially and temporally. Reliable reporting should confirm in-situ concentration sensors and OGI recordings, in accordance with OGMP 2.0 guidelines. Accurate quantification of leak rate through processing of OGI video sequences is a topic of great advances that will ultimately help us reduce greenhouse gas emissions.

## Figures and Tables

**Table 1:** Comparison of the key characteristics for the cooled and uncooled OGI cameras.

	Cooled MWIR	Uncooled LWIR
Sensor name	EOLE MW	PICO640S BB 7–14
Sensor technology	III–V HOT	VOx micro-bolometer
Spectral band	3.2–3.55 μm (cold filter)	6.5–14.0 μm + 7.0–8.75 μm (filter)
Image format/pitch	640 × 512/15 μm	640 × 480/17 μm
Sensitivity (typ.)	QE 70% ; NETD 22 mK (16 ms exposure)	NETD 19.2 mK; 85 mK (filtered)
Framerate	60 Hz	30 Hz
f-number N	f/1.3 (cold stop)	f/1.0 (lens)
Focal length (FL)	30 mm	35 mm
HFOV × VFOV	18.1° × 14.6°	17.7° × 13.3°

**Table 2:** Summary of KPIs for the cooled and uncooled thermal OGI cameras.

Camera	Cooled MWIR	Uncooled LWIR
Sensor	EOLE MW f/1.3	PICO640S BB 7–14
Filter	3.2–3.55 μm (cold)	7.0–8.75 μm (removable)
NETD/NETD*	22 mK/13.8 mK	85 mK/51.7 mK
NECL* at ΔT = 10 K	14 ppm·m	105 ppm·m
NECL* at ΔT = 5 K	31.6 ppm·m	223.6 ppm·m
MDLR* at 20 × 20 criterion	0.93 g/h	6.4 g/h
MDLR* at 9 × 5 criterion	0.35 g/h	2.5 g/h

**Figure A1:**
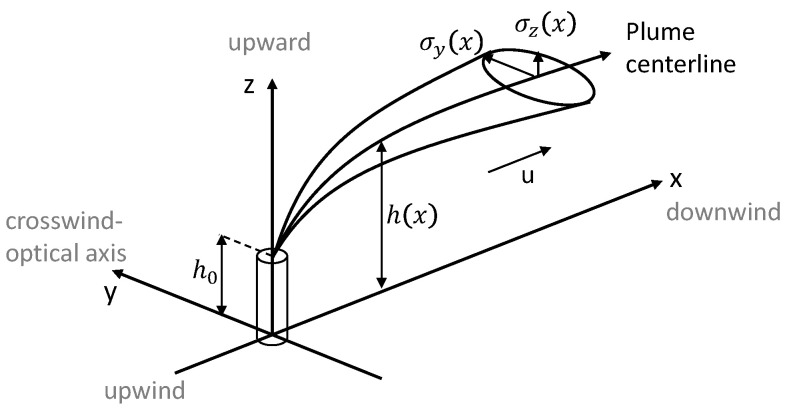
Geometry of the Gaussian plume model.

**Figure A2:**
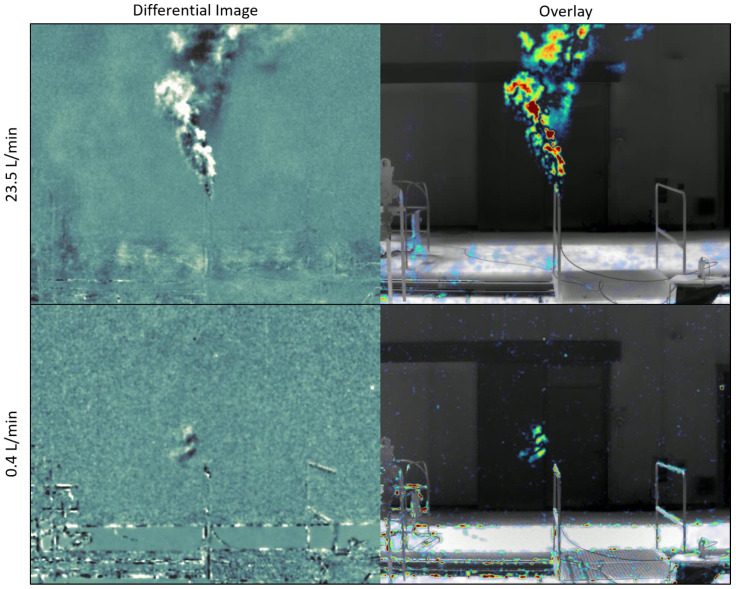
Controlled leak of natural gas viewed with the cooled camera: Differential (**left**) and overlay (**right**) representations. Large 23.5 L/min leak (**top**) and 0.4 L/min (**bottom**) leak rates.

## Data Availability

Raw data is not openly available due to non-disclosure agreements.

## Abbreviations

The following abbreviations are used in this manuscript:

BLIP	Background Limited Infrared Detector
CEMS	Continuous Emission Monitoring System
CH_4_	Methane
CL	Concentration Length, expressed in ppm·m
CNR	Contrast-to-Noise Ratio
DL	Digital Level
FL	Focal Length, expressed in mm
FOV	Field of View
IDCA	Integrated Dewar Cooler Assembly
IR	InfraRed
ISP	Image Signal Processor
KPI	Key Performance Indicator
LDAR	Leak Detection And Repair
LiDAR	Light Detection And Ranging
LoD	Limit of Detection
LWIR	Long-Wave InfraRed
MDLR	Minimal Detectable Leak Rate
MWIR	Middle-Wave InfraRed
NDIR	Non-Dispersive InfraRed
NECL	Noise Equivalent Concentration Length, expressed in ppm·m
NETD	Noise Equivalent Temperature Difference, expressed in mK
OD	Optical Depth, expressed in cm
OGI	Optical Gas Imaging
ppm	part per million ($10^{-6}$)
QE	Quantum Efficiency
q-OGI	quantitative OGI
SWIR	Short-Wave InfraRed
TDLAS	Tunable Diode Laser Absorption Spectroscopy
VGA	Video Graphics Array (640 × 512 or 640 × 480)

## Appendix A. Theory

### Appendix A.1. Concentration-Pathlength Absorbance Calculation

Let us consider a molecule of gas X (e.g., CH_4_). This molecule has different energy states, associated with the different modes of vibration of the coulombic bonds between their atoms. Absorption of a (infrared) photon of frequency $\nu_{i}$ (expressed as a wavenumber, in cm^−1^ units) sets the molecule into vibration, causing it to shift from energy $E_{i}$ to $E_{j}$, with the energy difference corresponding to the energy of the absorbed photon. In a low atmosphere (where atmospheric pressure prevails), such an energy transition ij is modeled by a Lorenz curve [9]. We define $C_{ij}\left(\nu \right)$ as the cross-section of the ij transition, which corresponds to a probability of the transition for a monochromatic wave at frequency ν (expressed as a wavenumber, in cm^−1^ units). It is homogeneous to a surface area and is expressed in cm^2^/molecule.

(A1) $$C_{ij}\left(\nu \right)=\frac{S_{ij}}{\pi }\frac{\gamma }{\gamma^{2}+{\left(\nu -\left(\nu_{ij}+\delta \right)\right)}^{2}}$$

$S_{ij}$ is the intensity of the transition at a reference temperature $T_{ref}=296$ K, expressed in cm^−1^/(molecule · cm^−2^). $\nu_{ij}$ is the photon frequency in the vacuum (expressed in wavenumber cm^−1^) associated with the energy gap between transition i and j. $\delta =\delta_{ref}p_{air}$ is the spectral shift of the transition, induced by the air pressure. We express it as a function of air pressure $p_{air}$ (in atm) and $\delta_{ref}$ (in cm^−1^/atm), the reference shift taken for 1 atm. Finally, γ is the half-width at half-maximum of the resonance: it represents the broadening of the transition induced by air. The HITRAN databe (https://hitran.org/) allows us to query a list of such transition parameters for a given molecule, in a given spectral interval. Let us take the example of methane, of chemical composition CH_4_, with all its isotopologue. Note that intensities $S_{ij}$ are weighted by isotopologue abundance in air. In the (500–5000 cm^−1^) spectral band, we retrieve from HITRAN a large number of transitions that we need to filter out the ineffective transitions down to 99.99% of the cumulative transition intensity. The absorption cross-section $C_{X}\left(\nu \right)$ of molecule X (in the CH_4_ example) is obtained by accumulating these transitions.

(A2) $$C_{X}\left(\nu \right)=\sum_{ij}C_{ij}\left(\nu \right)$$

We wish to convert such a cross-section spectrum into an absorbance that depends on the gas concentration (in ppm) and the pathlength (in m). The optical depth OD, a unitless quantity that quantifies the intensity of attenuation, is written by multiplying the molecular cross-section by the molecular density [X] (in molecules/m^3^) by the optical pathlength L (in m). We want to write this optical depth in the equivalent form, which involves $c_{X}$ the concentration in ppm and α(ν) the concentration-pathlength absorbance (expressed in ppm^−1^ · m^−1^).

(A3) $$OD\left(\nu \right)/L=\left[X\right]\cdot C_{X}\left(\nu \right)\cdot 10^{-4}=\alpha \left(\nu \right)\cdot c_{X}$$

The concentration $c_{X}$ is the ratio (in ppm) of the number of molecules X over the total number of air molecules. This can be written also as the ratio of the molecular volume densities. We can derive the molecular volume density from the perfect gas equation, and thus it depends on air pressure $p_{air}$ (in Pa) and air temperature $T_{air}$ (in K). $k_{B}$ is the Boltzmann constant.

(A4) $$c_{X}\left(ppm\right)=\frac{\left[X\right]}{\left[air\right]}10^{-6}=\left[X\right]\frac{k_{B}T_{air}}{p_{air}}10^{-6}$$

We rewrite α(ν) from the equivalence on the optical depth:

(A5) $$\alpha \left(\nu \right)=C_{X}\left(\nu \right)\cdot 10^{-4}\cdot \frac{\left[X\right]}{c_{X}}=C_{X}\left(\nu \right)\cdot 10^{-4}\cdot \left[air\right]\cdot 10^{-6}=C_{X}\left(\nu \right)\frac{p_{air}}{k_{B}T_{air}}\cdot 10^{-10}$$

### Appendix A.2. Description of the Gaussian Plume Model

We consider an outlet at the height $h_{0}$, which emits the pollutant (e.g., methane) at a rate Q (in g/s), and a horizontal wind in the x direction, at a wind-speed u (in m/s) as drawn in Figure A1.

The concentration profile of the pollutant along the plume centerline is assumed to have a Gaussian dispersion. The coordinate system is defined along the wind direction: with x, y, z being the downwind, crosswind, and vertical directions, respectively. The concentration map C(x,y,z) in g/m^3^ is expressed as the following.

(A6) $$C\left(x,y,z\right)=\frac{Q}{u}\frac{f(x,y)}{\sigma_{y}\left(x\right)\sqrt{2\pi }}\frac{g(x,y)}{\sigma_{z}\left(x\right)\sqrt{2\pi }}$$

where f(x,y) is the crosswind dispersion, which increases downwind:

(A7) $$f\left(x,y\right)=\exp\frac{-y^{2}}{2\sigma_{y}{\left(x\right)}^{2}}$$

g(x,z) is the vertical dispersion that takes into account reflection from the ground (but not from the ceiling):

(A8) $$g\left(x,y\right)=\exp\frac{-{\left(z-h\left(x\right)\right)}^{2}}{2\sigma_{z}{\left(x\right)}^{2}}+\exp\frac{-{\left(z+h\left(x\right)\right)}^{2}}{2\sigma_{z}{\left(x\right)}^{2}}$$

Gaussian deviation parameters $\sigma_{y}\left(x\right)$ and $\sigma_{z}\left(x\right)$ are expressed using the following equations:

(A9) $$\sigma_{y,z}\left(x\right)=\exp\left(I_{y,z}+J_{y,z}\ln x+K_{y,z}\ln^{2}\left(x\right)\right)$$

Coefficients $I_{y,z}$, $J_{y,z}$ and $Z_{y,z}$ are empirically expressed as a function of the atmospheric stability class proposed by F. Pasquill [49]. We consider typical laboratory conditions: wind-speed <2 m/s, daytime with slight solar radiation (<350 W/m^2^), which corresponds to Pasquill’s stability class B: ‘*moderately unstable*’. We recall those coefficients for this class:

**Table A1:** Pasquill’s stability coefficients (class B).

	$I_{y}$	$J_{y}$	$K_{y}$	$I_{z}$	$J_{z}$	$K_{z}$
Class B	−1.634	1.035	−0.0096	−1.999	0.8752	0.0136

The plume’s centerline height $h\left(x\right)=h_{0}+\Delta h\left(x\right)$ is expressed using empirical models, which depend on many environmental conditions. The plume rise Δh(x) is not a critical parameter in our OGI sensitivity model, so we will consider a very simple expression, valid for non-buoyant jet plumes, among the variety of those proposed by Briggs [50]. We consider gas temperatures close to air temperature (ΔT = 5 K), so buoyancy is neglected compared with wind-speed and exit velocity.

(A10) $$\Delta h\left(x\right)=2.3F_{m}^{1/3}u^{-2/3}x^{1/3}$$

The momentum flux parameter $F_{m}≃v_{g}^{2}r_{0}^{2}$ is given with the gas’ exit velocity $v_{g}$ (typ. 5 m/s) and the outlet radius $r_{0}$ (typ. 1/8’’).

## Appendix B. Image Examples

We show visualization methods, defined in Section 6, and applied with the cooled camera in the configuration as in Section 5 (suboptimal 25 mm f/2 lens), viewing a controlled leak of natural gas (80% methane) at a 9.5 m distance with clear weather, 29 °C ambient temperature and 40% relative humidity. The tests were performed at NaTran’s FenHYx test facilities (Alfortville, France). We show in Figure A2 two leaks at different rates: a large one at 23.5 L/min (about 0.9 kg/h), and a smaller one at 0.4 L/min (about 15.5 g/h), at the regulation level. Stronger denoising parameters are used in the smaller leak, similar to those used with the uncooled camera. The normalization step is not required thanks to a simpler background.

Another example is a view of a natural gas source: *La fontaine ardente*, translated ‘flaming fountain’ (Le Gua, France). Figure A3 is a snapshot taken with PICO640S BB 7–14 and a EFL 19 mm f/1.0 lens (from Umicore), at 2 m viewing distance, 21.7 °C ambient temperature, and 54.4% relative humidity. Due to the high contrast of the plume, mild denoising settings are applied, similar to that of the cooled camera.

The source burns continuously, but it was extinguished for the purpose of recording. Natural gas is invisible in the optical channel (Subfigure (a)). The hot stones stand out in the thermal channel (bright orange in Subfigure (b)), and so does the methane plume in both the differential (b) and overlay (c) display modes.
